# Compliance with Driver and Vehicle Licensing Agency guidance in a psychiatric inpatient setting

**DOI:** 10.1192/bjo.2021.215

**Published:** 2021-06-18

**Authors:** Nishanth Babu-Mathew, Sasha Chard, Sarah Winfield

**Affiliations:** Merseycare NHS Foundation Trust

## Abstract

**Aims:**

Driving is complex, requiring adequate: attention and concentration, memory, insight and understanding, judgement, planning and the ability to self-monitor1. Psychiatric illness, and associated medications, may affect patients’ ability to drive safely. The DVLA is responsible for determining individuals’ safety to drive and produces guidance specific to psychiatric disorders. Patients must comply with relevant guidance and clinicians must determine patients’ driving status and offer appropriate advice about medications and any need to inform the DVLA. This audit aimed to determine the compliance with DVLA guidance on a single inpatient psychiatric ward within Merseycare NHS Foundation Trust, UK.

**Method:**

A retrospective review of electronic patient records was completed. Clerical staff identified all patients admitted to Windsor House from 1/8/20–30/11/20 (n = 42). Data relating to driving status and driving advice were collected onto individual patient audit proformas, and uploaded to the online Audit Management and Tracking (AMaT) system.

**Result:**

100% of patients had diagnoses that would require the DVLA to be informed and 100% were prescribed medication with potential side effects that could impair ones’ ability to drive safely such as dizziness, drowsiness or impaired concentration2. Driving status was only documented for 12 patients (29%) and type of vehicle driven for only 6 patients (1 of whom had an HGV licence).

Discussion of DVLA guidance within the last 3/12 by the mental health team was documented in 17% patients. Of these patients, appropriate driving advice was given to 86%. All patients advised to cease driving were willing to. No patients were advised about side effects of medications on driving. No notes evidenced if the DVLA had been informed of patients’ admission, diagnosis or medication regimes.

**Conclusion:**

Discussing diving status and DVLA advice with psychiatric patients is important but may not always happen in inpatient settings, despite most patients having a relevant diagnosis. Failure to determine driving status may mean some patients are not being given appropriate guidance as required. Counselling on medication side effects in relation to driving should be encouraged as the majority of patients are taking prescribed medication that can potentially impair driving. Recommendations to improve compliance include: adding “driving status” to admission clerking and ward review proformas, educating staff to actively discuss driving with inpatients and create discharge checklists which prompt discussing driving status, medications and driving advice, and to re-audit in 6 months time.

